# Electrochemotherapy of Tumours

**DOI:** 10.3791/1038

**Published:** 2008-12-15

**Authors:** Gregor Sersa, Damijan Miklavcic

**Affiliations:** Department of Experimental Oncology, Institute of Oncology Ljubljana; Faculty of Electrical Engineering, University of Ljubljana

## Abstract

Electrochemotherapy is a combined use of certain chemotherapeutic drugs and electric pulses applied to the treated tumour nodule. Local application of electric pulses to the tumour increases drug delivery into cells, specifically at the site of electric pulse application. Drug uptake by delivery of electric pulses is increased for only those chemotherapeutic drugs whose transport through the plasma membrane is impeded. Among many drugs that have been tested so far, bleomycin and cisplatin found their way from preclinical testing to clinical use. Clinical data collected within a number of clinical studies indicate that approximately 80% of the treated cutaneous and subcutaneous tumour nodules of different malignancies are in an objective response, from these, approximately 70% in complete response after a single application of electrochemotherapy. Usually only one treatment is needed, however, electrochemotherapy can be repeated several times every few weeks with equal effectiveness each time. The treatment results in an effective eradication of the treated nodules, with a good cosmetic effect without tissue scarring.

**Figure:**
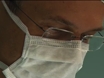


## Protocol

The treatment procedure for electrochemotherapy consists of local or systemic drug injection followed by delivery of electric pulses applied to the tumour ^1^. Detailed information about the treatment is published in Standard Operating Procedures (SOP) ^2^.

### Treatment indications:

Tumour type: basal cell carcinoma, and cutaneous metastases of malignant melanoma, breast carcinoma, hypernephroma, Kaposi's sarcoma and others.
Tumour location: accessible cutaneous and subcutaneous tumour nodules on all parts of the body.
Tumour size can be from 3 mm to 3-5 cm in diameter.
Number of nodules; from 1 to up to 100.

### Treatment procedure:

Local or general anaesthesia, depending on the number of the treated nodules and the location.
Drug choice: cisplatin or bleomycin. Cisplatin and bleomycin can be injected intratumorally, bleomycin also intravenously in a dose of 15000 IU/m^2^. Injected dose for intratumoural application of bleomycin is 250-1000 IU/cm^3^ of tumour tissue and for cisplatin 0.5 -1 mg/cm^3^ of tumour tissue
Time interval between the drug administration and application of electric pulses; after intratumoral injection of the drug immediately, after intravenous administration after 8 min. up to 28 min.
Electric pulse generator for electrochemotherapy should provide square wave electric pulses of amplitude 1000 V or more, repetition frequency from 1 Hz to 5 kHz.
Electric pulse parameters: 8 pulses of e.g. 1000 V at 8 mm distance between two plate electrodes, frequency 1 Hz or 5 kHz, duration of the pulse 100 µs.
If the whole tumour can not be encompassed between the electrodes, application of electric pulses should be repeated that many times so as to cover the whole tumour area, including safety margins.
Electrode type: plate electrodes are used for small, superficial tumour nodules, needle electrodes are for treatment of deeper seeded nodules under the skin.

### Other features of the treatment:

Usually single treatment is effective for tumour nodule eradication; however electrochemotherapy can be repeated in 4 weeks intervals on the remaining tumour tissue or other tumour nodules.

Due to lack of systemic toxicity - because very low doses of bleomycin or cisplatin are needed - patients can be treated on an out-patient basis, and can leave the hospital soon after the treatment.

## Discussion

Increase in cytotoxicity of bleomycin by exposure of cells to electroporative electric pulses was first described by Okino M and Mir LM ^3,4^. Thereafter electrochemotherapy was demonstrated to be effective also for cisplatin ^5^. Extensive preclinical data were collected on in vitro and in vivo tumour models in the following years. Treatment effectiveness was determined in relation to drug dosage, route of its administration, timing of drug injection and application of electric pulses, intensity of the electric field, coverage with sufficiently high electric field (E), and appropriate selection of electrodes and positioning with respect to the tumour ^1,6,7,8^. Furthermore, mechanisms underlying the effectiveness of electrochemotherapy were elaborated, demonstrating that, besides direct effect of electrochemotherapy to tumour cells, vascular disrupting effect and immune response are involved ^1,6,9^. All of these collected data enabled translation of electrochemotherapy into the clinics.

The first clinical trials demonstrated effectiveness of electrochemotherapy on head and neck and melanoma tumour nodules ^10^. Later on, its effectiveness was demonstrated on other tumour types, such as basal cell carcinoma of the skin, cutaneous metastases of melanoma, mammary tumours, hypernephroma and Kaposi's sarcoma. There are several reports evaluating collectively all clinical data published on electrochemotherapy with bleomycin and cisplatin ^11-15^. Overall, the response rate of the treated tumours was approximately 80% objective responses and approximately 70% complete responses ^15,16^. The effectiveness can be even higher by repetitive treatment ^17^.

All of these clinical data have enabled electrochemotherapy to be adopted in some European countries as standard treatment, with palliative intent, on various tumours. The future of this treatment is to introduce electrochemotherapy in treatment of internal tumours and metastases, and in combined treatment, either with gene therapy or radiation therapy. These attempts are already under way.

